# Twenty Element Concentrations in Human Organs Determined by Two-Jet Plasma Atomic Emission Spectrometry

**DOI:** 10.1155/2019/9782635

**Published:** 2019-10-24

**Authors:** Natalia P. Zaksas, Svetlana E. Soboleva, Georgy A. Nevinsky

**Affiliations:** ^1^Nikolaev Institute of Inorganic Chemistry, Siberian Division of Russian Academy of Sciences, Pr. Akademika Lavrentieva 3, Novosibirsk 630090, Russia; ^2^Institute of Chemical Biology and Fundamental Medicine, Siberian Division of Russian Academy of Sciences, Pr. Akademika Lavrentieva 8, Novosibirsk 630090, Russia

## Abstract

In this paper, we have performed determination of the concentration of twenty elements in seven human organs (spleen, liver, kidney, muscle, heart, lungs, and brain) using two-jet plasma atomic emission spectrometry. The method allows multielemental analysis of solid samples without wet acid digestion. Before analysis, all human organs were first dried, ground to powders, and carbonized. The relative content of elements in each of the seven organs was very different depending on the donor. The average content of twenty elements in various organs varied in the following ranges (*μ*g/g of dry weight): Ag (<0.02–0.2), Al (2.1–263), B (<0.5–2.5), Ca (323–1650), Cd (<0.1–114), Co (<0.2–1.0), Cr (<0.5–4.0), Cu (4.2–47), Fe (156–2900), Mg (603–1305), Mn (0.47–8.5), Mo (<0.2–4.9), Ni (<0.3–3.1), Pb (<0.3–1.9), Si (31.6–2390), Sn (<0.3–3.2), Sr (0.2–1.0), Ti (<2–31, mainly in lungs), and Zn (120–292). The concentration range of Ba in organs of five donors was <0.2–6.9 and 2.0–5600 for one donor with pneumoconiosis (baritosis). The maximum element contents were found, respectively, in the following organs: Al, B, Cr, Ni, Si, Sn, Sr, Ti (lungs), Fe (lungs and spleen), Mn (liver and kidney), Ag and Mo (liver), Ca (lungs and kidney), Cu (brain), Cd (kidney), Pb (brain), and Zn (liver, kidney, and muscle). The minimal content of elements was observed, respectively, in the following organs: Ag (all organs except liver), Ba (spleen, muscles, and brain), Ca and Mg (liver), Si (liver, muscle, and brain), Cd and Sr (heart and brain), Al, Cu, Fe, and Mn (muscle), and Zn (spleen and brain). The analysis of possible biological role and reasons for the increased content of some elements in the organs analyzed was carried out.

## 1. Introduction

Microelements play different essential roles in many physiological processes in all biological systems and organs in both normal physiological and pathological conditions [1–10]. They participate in regulating temperature, transporting nutrients and gases, maintaining acid-base balance, maternal and child mental health, homeostasis of the human organisms, the functioning of enzymes, protein, and DNA syntheses, cytoskeleton activation, etc. The content of various elements, including metal ions in different organs, tissues, and biological fluids, can vary in a wide range. To understand the features of their functioning, the relative content of different elements in organs and tissues must be analyzed. For the analysis of biological samples, atomic absorption spectrometry (AAS), inductively coupled plasma atomic emission spectrometry (ICP-AES), and mass spectrometry (ICP-MS) are widely used [11, 12]. These methods require sample digestion in mixtures of different acids (HNO_3_, HClO_4_, H_2_O_2_, etc.) using high pressure, microwaves, ultraviolet, or ultrasound. The samples of 200–300 mg, which are not always available, are usually needed. Direct analysis, with little or without prior chemical treatment, is desirable because of the simplicity of the analytical procedure and reduction of the risk of contamination and analyte loss. Combination of electrothermal atomization and vaporization with the above methods allows analysis of solid biological samples, but different behavior of the analytes during evaporation, poor reproducibility, and calibration requirements make it difficult to use these methods for routine multielemental analysis [13–19]. Using laser ablation ICP-MS [20, 21], X-ray fluorescence spectrometry [22, 23] and neutron activation analysis (NAA) [24] for direct analysis of solid biological samples are also complicated by lack of certified reference materials (CRMs) with different biological matrices. In addition, NAA is not usually available in analytical practice.

**Figure 1:**
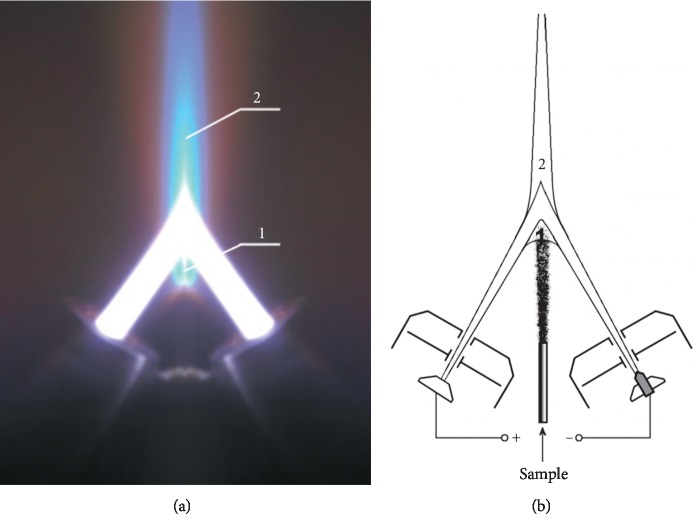
Plasma torch (a); electrode unit and analytical regions of the plasma flow (b): 1, before the jet confluence; 2, after the jet confluence.

In this work, two-jet plasma atomic emission spectrometry (TJP-AES) was used. The method was developed in the mid-70s [25]; the present study was performed using a new Plasmatron designed at “VMK-Optoelektronika” (Russia). The plasma torch photograph and scheme of electrode unit are presented in Figure 1. The TJP power is by an order of magnitude higher than the ICP one, which allows direct analysis of powdered samples. The possibilities of TJP-AES for the analysis of powdered biological samples [26], dry plasma [27] and whole blood [28], bone [29], and animal organs [30] without wet acid digestion were demonstrated. Comparatively, weak matrix effects in the TJP make it possible to apply the same calibration samples based on graphite powder for analysis of these samples. The techniques, direct and after sample carbonization, were offered. 5–10 mg dry sample is quite enough for direct analysis, and about 50 mg is needed for carbonization. The possibility of analysis of small amount samples and usage of unified calibration makes TJP-AES promising for biomedical investigations.

The method was used for the analysis of the plasma of 2-month-old balb/c mice (control group) and mice treated with CoCl_2_ [31]. It was shown that the content of Ca, Cu, and Zn did not appreciably change while significant increase in the content of Si (3.2-fold), Fe (2.0-fold), and Mg (1.4-fold) was found.

The metal concentrations were also measured in the samples of lyophilized plasma of healthy Wistar rats and homogeneous IgGs separated from the plasma [27]. It was found that the relative amount of different metals decreased in the order: Ca > Mg > Fe > Cu ≥ Zn > Al ≥ Sr > Ti ≥ Mo ≥ Mn > Ni and Fe ≥Pb ≥ Zn ≥ Cu ≥ Al ≥ Ca ≥ Ni ≥ Mn > Co ≥ Mg in the plasma and IgGs, respectively.

It was shown in many articles that polyclonal IgGs from sera of healthy volunteers and experimental mice can catalyze different chemical reactions (for review, see [32–36]). The occurrence of autoantibodies with catalytic activities is a distinctive feature of mammalian autoimmune diseases [32–36]. It is known that metal ions can play an important role in the functioning of a considerable number of different enzymes and proteins. It was shown that, in the absence of metal ions (in the presence of EDTA), antibodies from autoimmune mammals were usually catalytically inactive, but they catalyzed different chemical reactions after the addition of external metal ions: peroxidase (H_2_O_2_-dependent) and oxidoreductase (H_2_O_2_-independent) oxidation of substrates, hydrolysis of DNA, RNA, proteins, and peptides [32–36]. Thus, TJP-AES is a reasonably universal approach to the evaluation of different elements in a variety of samples.

In the present study, we have done the first analysis of twenty elements in seven human organs by TJP-AES.

## 2. Materials and Methods

### 2.1. Materials

Samples of six organs (spleen, liver, kidney, muscles, heart, and lungs) were taken from five previously healthy donors who died in various types of disasters and one deceased person with pneumoconiosis (baritosis). The only sample of brain was taken from the donor (number 4) who died due to a traumatic brain injury. The study was approved in accordance with the law of the Russian Federation including the written consent of donors' agreement in the period before their injury when they were still alive (or their relatives' after death) to submit small fragments of their organs after death for scientific purposes according to the guidelines of the Helsinki Ethics Committee.

### 2.2. Preparation of Samples and Element Content Determinations

Small fragments of organs (about 50–100 g) were frozen at −70°C; then, they were thoroughly dried by lyophilization and ground. To get fine powders, carbonization was applied. This procedure makes the tissues more fragile, which allows getting powders with particle size 20–30 *μ*m after grinding in a Plexiglas mortar. Moreover, preconcentration of elements takes place, which provides lower their detection limits. For the carbonization procedure, a100–150 mg freeze-dried sample was placed in a previously weighed quartz cup, put into a quartz electric resistance furnace at room temperature, and heated at 250°C for 15 min and then at 400°C for 15 min. The remainder was weighed, ground in a Plexiglas mortar, and diluted with a spectroscopic buffer (graphite powder containing 15 wt. % NaCl) 10 and 100 times. A 100-fold dilution is needed for determining high element concentrations to avoid analytical signal oversaturation. 15–20 mg of diluted sample was introduced into the plasma.

Graphite powder containing15 wt. % NaCl with the impurity concentration range of 0.01–100 *μ*g/g was used to obtain calibration curves. These samples were prepared from Russian State Certified Reference Material of graphite powder with different known compositions of impurities (SOG-24 and SOG-37 containing 24 and 37 impurities, respectively; Ural State Technical University).

The high-power two-jet plasmatron (10–12 kW) was used. The analysis was performed under the following conditions: current strength 85 A, plasma gas (argon) 4 l/min, carrier gas 0.7 l/min, angle between jets 60°, and analytical region 4-5 mm lower than the point of the jet confluence. A diffraction spectrograph with a 2400 lines/mm grating covering two spectral ranges (185–350 and 390–460 nm) was used. Spectrum registration was performed using a multielement photodiode analyzer of emission spectra produced by “VMK Optoelektronika” (Russia).

### 2.3. Statistical Analysis

The average values of metal concentrations (mean ± SD) were estimated using three independent assays for each sample of all organs analyzed. The criterion of Shapiro–Wilk Test was used to check the normality of distribution. Several of the sample sets did not fit the Gaussian distribution. In this case, the median (*M*) and interquartile ranges (IQR) were also estimated.

## 3. Results

Analysis of seven freeze-dried human organs (lung, spleen, liver, kidney, muscle, heart, and brain) ground to powders was performed using TJP-AES. The content of twenty elements was estimated (Supplementary Tables [Supplementary-material supplementary-material-1]–[Supplementary-material supplementary-material-1]). It was possible to take the sample of the brain only from one donor (number 4) who died after a traumatic brain injury. The element concentrations in different organs of donor number 4 are given in Table 1. The average values of element concentration in various human organs of all donors are given in Table 2. Al, Ca, Cu, Fe, Mg, Mn, Si, Sr, and Zn are present in well-detectable amounts in all organs of the donors. According to the average values of concentration, they showed the maximum content of Fe, Mg, and Ca for all organs analyzed. The highest and comparable average concentration of iron was observed in the lung (2900 *μ*g/g) and spleen (2650 *μ*g/g), and it was significantly smaller in other organs (*μ*g/g): kidney (825) > liver (723) > brain (440) ≥ heart (428) > muscle (156). The relative content of magnesium was to some extent comparable in all organs (968–1305 *μ*g/g), except liver (603 *μ*g/g). The average calcium content decreased in the following order (*μ*g/g): lung (1650) > kidney (1232) > spleen (511.7) > muscle (442) ≥ brain (430) ≥ heart (413) ≥ liver (323). The relative content of silicon in the lungs (2390 *μ*g/g) was approximately 15- to 76-fold higher than in other organs (31.6–164 *μ*g/g) (Table 2).

Zinc content in all organs varied in the range 120–292 *μ*g/mg. The concentration of aluminum in the lungs (263 *μ*g/g) was about 18- to 125-fold higher than in other organs (2.1–14.5 *μ*g/g) (Table 2). The copper content varied in the range 4.2–47 *μ*g/g, and it was maximal in the brain and minimal in muscles.

In the case of cadmium, a completely different situation was observed. The maximum content of cadmium was found in kidney (114 *μ*g/g), and it was 10–1140 times higher than the content in other organs (<0.1–11.5 *μ*g/g) with minimal concentration in lungs and heart of some donors (<0.1 *μ*g/g; Supplementary Tables [Supplementary-material supplementary-material-1] and [Supplementary-material supplementary-material-1]).

The maximum content of manganese was found in the liver and kidney (7.9–8.5 *μ*g/g), while in other organs its concentration was significantly lower (0.47–2.7 *μ*g/g). The content (*μ*g/g) of titanium (31 lungs and <2–2.5 other organs), chromium (4.0 and <0.5), tin (3.5 and <0.5), and boron (2.5 and <0.5–0.95) in the lungs was also higher than in other organs (Table 2). In contrast, maximum molybdenum content was observed in the liver (4.9 *μ*g/g). All organs contained strontium (0.2–1.0 *μ*g/mg) and silver (<0.02–0.2 *μ*g/mg) at relatively low concentrations (Table 2).

We had six organs from five deceased healthy donors and one deceased patient with occupational pneumoconiosis (baritosis, donor number 2 in Supplementary Tables [Supplementary-material supplementary-material-1]–[Supplementary-material supplementary-material-1]).  Table 3 demonstrates that, in all organs of donors 1 and 3–7, the relative content of Ba is relatively low (<0.2–2.8 *μ*g/g) except lungs (1.9–21 *μ*g/g). At the same time, the content of Ba in lungs, liver, and muscle of donor with baritosis is 267- to 2950-, 46- to 650-, and 367-fold, respectively, higher than that for other donors (Table 3). The difference in the content of Ba in other organs is somewhat less (fold): spleen (≥130), kidney (9–18), and heart (2.9–13).

## 4. Discussion

In this paper, we estimated for the first time the content of twenty elements (mostly metals) in six different human organs. The mass content of all metals in the human body is approximately 2.5% [37]. Fraction of mass in human organisms of the two analyzed metals Ca (1.4 × 10^−2^) and Mg (5 × 10^−4^) belong to the macro, while eighteen other to microelements, and their mass content in the human body is significantly different (fraction of mass of human organisms): Fe (6.0 × 10^−5^), Zn (3.2 × 10^−5^), Mo (2.0 × 10^−5^), Sr (4.6 × 10^−6^), Pb (1.7 × 10^−6^), Cu (1.0 × 10^−6^), Al (8.7 × 10^−7^), Cd (7.2 × 10^−7^), B (6.9 × 10^−7^), Ba (3.1 × 10^−7^), Sn (2.4 × 10^−7^), Mn (1.7 × 10^−7^), Ni (1.4 × 10^−7^), Ti (1.3 × 10^−7^), Si (1.2 × 10^−7^), Cr (2.4 × 10^−8^), Co (2.1 × 10^−8^), and Ag (1.0 × 10^−8^) [37–43].

To carry out the estimation of the content of various elements in different organs, we did not remove blood before their lyophilization. The blood accumulates various elements and partly the content of elements in the organs is determined by their content in blood (*μ*g/g of blood): Fe (745–1050), Mg (36–64), Ca (8–31), Zn (8.0–14.5), Pb (<0.9), Cu (0.52–0.89), Se (0.19–0.38), Ni (0.001–0.3), Mn (0.007–0.03), Mo (0.005–0.02), and Co (<0.001) [44], as well as (g/ml): total metals (0.20–0.25), Fe (6.0 × 10^−5^), Mg (3.2–5.5 × 10^−5^), Ca (4.5–4.9 × 10^−5^), Mo (2 × 10^−5^), Sr (4.6 × 10^−6^), Zn (3.5 × 10^−6^), Pb (1.7 × 10^−6^), Cu (7–16 × 10^−7^), Al (8.7 × 10^−7^), B (6.9 × 10^−7^), Ba (3.1 × 10^−7^), Mn (0.1–2.5 × 10^−7^), Ni (1.4 × 10^−7^), Ti (1.3 × 10^−7^), Si (1.2 × 10^−7^), Co (2.1 × 10^−8^), Ag (1.0 × 10^−8^), and Cd (1.5 × 10^−9^) [45]. From the ratio of the content of metals in various organs and blood, one can see that the relative content of various elements in the human body as a whole and in the blood differs significantly.

The content of Al, Ca, Cd, Co, Cu, Fe, Mg, Mn, Mo, and Zn in seven organs was evaluated previously in Japanese donors by neutron activation analysis [46].

A variety of metal-dependent enzymes and proteins are part of all the organs analyzed by us. More than 25% enzymes contain strongly bound some of the metal ions (more often Mg^2+^, Ca^2+^, Fe^2+^, Zn^2+^, Cu^2+^, Mn^2+^, Co^2+^, and Ni^2+^), and these enzymes are active only in the presence of specific metal ions [47, 48]. In addition, metal ions are components of many hormones and vitamins; some detected metal ions play other very different biological functions in the organs used for the study.

The biological role of Al is poorly understood. It is supposed that Al is not required for any biological processes, but it possesses very low toxicity [49]. The average values ± SD of the Al content in various organs obtained in our work and in [46] agree well for all organs except muscle (2.2 ± 1.8 and 9.4 ± 2.3 *μ*g/mg, respectively) and brain (2.1 and 13.9 ± 3.2 *μ*g/g) (Table 2).

Calcium ions are necessary for hematopoiesis, the coupling of cells with each other ones, metabolism, reduction of vascular permeability, normal growth of the skeleton, and normal state of the nervous system, and they have an anti-inflammatory effect [50]. All these functions of calcium are undoubtedly important for the functioning of all human organs. The Ca content is very high in all organs analyzed (average values: 323–1650 μg/g of dry weight) (Table 2). The average calcium content (*μ*g/g) found by us in Russia's dead people is in average higher than in Japan's ones [46] in lungs (1650 ± 565 and 585 ± 205, respectively) and in kidney (1232 ± 410 and 529 ± 291).

Cadmium is a nonessential and toxic element for humans [50, 51]. The concentration of Cd was also estimated by ICP-AES in human kidney of 47 patients: according to [52], it varies in the range of 37–732 *μ*g/g of dry tissue, which is to some extent comparable to the values found by us: range 9.3–290 and average value 114 ± 122 *μ*g/g of dry tissue (Table 2 and Supplementary [Supplementary-material supplementary-material-1]). The ranges of possible concentrations of Cd in Russian and Japanese [46] samples of various organs do not contradict to each other.

Cobalt is known as an essential element for mammals [50, 53]. Cobalt performs a variety of functions, as it forms the catalytically active centers of enzymes necessary for synthesis of DNA and the metabolism of amino acids. The Co content in several Russian people (Table 2) compared with the Japanese ones [46] may be higher (*μ*g/g): in liver (<0.2–1.0 and 0.17 ± 0.11, respectively), kidney (<0.2–0.38 and 0.07 ± 0.1), and heart (<0.2–0.74 and 0.07 ± 0.048).

The ranges of Cu, Mg, and Mn content in different organs of the Russian and Japanese groups of samples are comparable. We find the increased concentration of Cu in preparations of the liver, kidney, heart, and lungs (average content 15.2–19.8 *μ*g/g) and the highest content in one preparation of brain (47 *μ*g/g; Table 2).

Cu^2+^ promotes plastic metabolism, accelerates the recovery of muscle mass, is involved in the synthesis of collagen and elastin, promotes the elasticity of the lungs, skin, and blood vessels, helps in the functioning of the central nervous system, hair and skin pigmentation, and also strengthens the nervous system [50]. Increased content of Cu^2+^ in the brain can be associated with the formation and realization of the functions of the brain nervous system. Using AAS, it was shown that the average content of Cu in human liver is 14.2 ± 7.0 *μ*g/g (dry weight) [54], which agree with our data 19.8 ± 5.7 *μ*g/g (Table 2). The content of copper in organs of the Russian and Japanese samples [46] is in good agreement (Table 2).

The average content of Mg^2+^ ions is also high in all organs (603–1305 *μ*g/g; Table 2). Perhaps, this is the consequence of the fact that Mg^2+^ is the most common cofactor of various enzymes and proteins, and it is important not only for stimulating the catalysis of various chemical reactions but also for achievement of optimal conformations of proteins, nucleic acids, and other substrates [47, 48]. In addition, Mg^2+^ has an antiseptic and vasodilating action that suppresses blood pressure and cholesterol in the blood and has a calming effect on the nervous system. The decrease in Mg^2+^ in bones leads to the development of osteoporosis. The magnesium content in six organs found by us and in Japanese samples [46] is well consistent (Table 2).

The relative content of Mn was estimated in the human brain of an older woman by graphite furnace AAS; in different parts of the brain, it varied from 0.44 to 4.8 pg/g wet tissues [55]. It is difficult compared with dry powder of the brain (1.8 *μ*g/g) of one sample used in our work (Table 1). Mn content estimated by AAS in dry liver (with one exception corresponding to 12.9 *μ*g/g) ranged 0.22–4.6 *μ*g/g with an average mean of 2.3 ± 1.0 *μ*g/g [54]. The content of manganese in our liver samples demonstrated average value 8.5 ± 2.0 *μ*g/g (Table 2) and varied in the range 5–10 *μ*g/g (Supplementary [Supplementary-material supplementary-material-1]). The content of manganese in the six organs of our groups and Japanese groups [46] is overall, to some extent, comparable (Table 2).

Human body Mo plays a major role, without which it is impossible to carry out many processes [50, 56]. Molybdenum in the body participates in oxidative processes, triggering the transition of oxygen and nutrients into energy, which is necessary to maintain the work of tissues and cells. If molybdenum is present in minimal amounts, the oxidative enzymes are practically not active, which leads to problems with human health [56]. The relative content of Mo in various organs decreases in the following order: liver > kidney > spleen > heart > lungs, but its concentration was shallow in muscle and brain (Table 2). Some Japanese samples [46] demonstrated higher content of Mo (*μ*g/g): lungs (0.33 ± 0.06 our group and <0.01–2.7 Japanese one), muscle (<0.2 and <0.31–2.2), heart (<0.2–0.55 and 0.14–3.9), and brain (<0.2 and <0.02–1.65). Mo concentrations in liver and kidney are comparable.

The highest concentration of Fe is found in lungs (average content 2900 *μ*g/g) and spleen (2650 *μ*g/g). The blood contains iron-containing ferritin and transferrin in a high concentration. The primary role of lungs is to bring oxygen from the atmosphere and pass it using ferritin and transferrin into the bloodstream. Therefore, the content of iron ions bound to the proteins in the lungs should be increased. The spleen acts primarily as a blood filter [57]; therefore, its content of Fe ions may be higher than in other organs (Table 2). The kidney receives blood from the paired renal arteries, which goes into the paired renal veins. Kidneys change blood plasma by filtration, reabsorption, secretion, and excretion; one-fifth of the blood volume entering the kidneys is usually filtered [58]. Thus, it is not surprising that the kidney also contains Fe ions in a high concentration (average value 825 *μ*g/g, Table 2). Heart pumps blood containing proteins bound with Fe ions through the vessels of circulatory system; therefore, it also contains Fe in relatively high concentration (average value 428 *μ*g/g, Table 2). Estimated by us, the average iron content was higher than in [46] in four organs (*μ*g/g): lungs (2900 ± 498 and 984 ± 489, respectively), spleen (2650 ± 1595 and 1400 ± 760), kidney (825 ± 299 and 430 ± 203), and heart (428 ± 222 and 257 ± 80).

Interestingly, four organs (liver, kidney, muscle, and heart) in average contain Zn in comparable concentrations (218–292 *μ*g/g, Table 2). The blood supplies the liver by two routes, hepatic artery (20–40%) and the portal vein (60–80%); it receives blood approximately 1.5 l/min [59]. Liver cells contain thousands of different enzymes, including zinc-containing carbonic anhydrase, various oxidases, and other Zn^2+^-dependent enzymes, which are responsible for the metabolism of components harmful to humans. However, zinc ions and zinc-dependent enzymes from the liver are carried by blood to all other organs. Taking into account the range of the values, the average Zn content in our group was significantly higher than that of the Japanese group [46] only in the case of lungs (208 ± 35 and 62 ± 19 *μ*g/g). Zn content in the liver ranged from 38.5 to 231.3 *μ*g/g, and the average value was 118.3 ± 44.4 *μ*g/g [54]. The zinc content in the liver of the donors studied by us was noticeably higher: range 140–520 *μ*g/g and average value 272 ± 153 *μ*g/g (Supplementary [Supplementary-material supplementary-material-1]).

At the same time, the content of ten elements was determined by us for the first time: Ag, B, Ba, Cr, Ni, Pb, Si, Sn, Sr, and Ti (Tables 1 and 2 and Supplementary Tables [Supplementary-material supplementary-material-1]–[Supplementary-material supplementary-material-1]). These 10 elements in different human organs can have very diverse biological roles.

Silver is stored mainly in the skin and liver in smaller amounts than in other organs [60]. Like most heavy metals, this element is not very important, but similar to all heavy metals entering the body, exhibits a toxicity effect [60]. Ag was found in relatively low and comparable concentrations in all organs (<0.02–0.06 *μ*g/g; Table 2) except the kidney of one donor (0.11 *μ*g/g) (Supplementary [Supplementary-material supplementary-material-1]).

Boron is a chemically dynamic element forming approximately 230 compounds, generally with other elements [61]. The average content of B is approximately the same in all organs (0.6–1.0 *μ*g/g) except lungs (2.5 *μ*g/g) (Table 2).

Barium has been associated with a number of adverse health effects in both humans and experimental animals [62]. Both human and animal evidence suggests that the cardiovascular system may be one of the primary targets of barium toxicity. In addition to cardiovascular effects, exposure of humans and/or animals to barium has been associated with respiratory, gastrointestinal, hematological, musculoskeletal, hepatic, renal, neurological, developmental, and reproductive effects. However, the maximum average concentration of Ba was detected by us in the lungs (6.9 *μ*g/g) of five donors and decreased in the following order (*μ*g/g): liver (<0.2–2.) > heart (0.36) > kidney (<0.2–0.40) > muscle (∼0.3) ≈ spleen (<0.3) ≈ brain (<0.3) (Table 2). One of the six donors (donor (2) suffering from baritosis showed very high concentrations of barium in lungs (5600 *μ*g/g) and in other organs: liver (130), muscles (110), spleen (39), kidney (3.5), and heart (2.0) (Table 3). The extremely high Ba concentration in the lungs of donor 2 may be due to prolonged inhalation of dust containing Ba compounds. Overstated results by one or more than two orders of magnitude were obtained for other organs (Table 3). In addition, an increased Sr content is observed in the lung of donor 2 (Supplementary [Supplementary-material supplementary-material-1]), which can be because of the fact that Sr accompanies Ba in nature, and in all probability, it was contained in the dust.

Chromium maintains a normal level of glucose, enhances the action of insulin, regulates lipid metabolism, ensures the structural integrity of nucleic acids, regulates the thyroid glands, and regulates the activity of the heart muscles [63]. The content of chromium in all organs is lower than the detection limit of the technique (<0.5 *μ*g/g), except for the lungs (4.0 *μ*g/g) (Table 2).

Ni^2+^ has complexes with amino acids, carboxylic acids, and other biologically active compounds that are donors of N- or O-groups [53]. Owing to many complex formations, nickel stimulates the synthesis of amino acids in the cells, accelerates the regeneration of blood plasma proteins, and normalizes the content of hemoglobin in the blood. However, the content of Ni in all organs (*μ*g/g) was relatively low (<0.3–0.5) except the lungs (3.1) and kidney of some donors (0.83–2.5; Supplementary [Supplementary-material supplementary-material-1]).

The biological role of certain human trace elements, including Pb in low concentrations, is not yet fully understood. However, in high concentrations, Pb is toxic elements. Pb affects the body's cells, destroying the protein [64]. This metal binding to the protein disulfide group provokes the destruction of the tertiary structure of the protein (denaturation). This is considered as a key cause of their death and the occurrence of inflammatory processes. Pb was revealed in relative low and comparable concentrations in all organs (<0.3–0.65 *μ*g/g) except the kidney of one donor (1.7 *μ*g/g; Supplementary [Supplementary-material supplementary-material-1]) and brain of donor number 4 (Table 1).

Silicon plays a fundamental role in the constitution and balance of immune system of the body, and in all cells, it plays a protective role [65]. The mammalian bodies cannot absorb calcium without the presence of silica. Therefore, silicon is necessary in all organs of humans. As several other elements (see above), the increased concentration of Si was revealed in lungs (2390 *μ*g/g) and in lower concentration in other organs: spleen > kidney > heart > brain > muscle (Table 2).

Sn activates the growth of tissues and organs, responsible for the proper formation and development of the skeleton; cofactor of gastric enzyme gastrin, important for the production of bile acids and flavin enzymes [66]. As in the case of some other elements, the content of tin in various organs is low and comparable (<0.3–1.1 *μ*g/g) except for lungs (3.2 *μ*g/g) (Table 2).

The biological role of strontium is not exactly known. But the uptake of high strontium concentrations is generally not known to be a great danger to human health [67]. In one case, someone experienced an allergic reaction to strontium, but there have been no similar cases since. For children, exceeded strontium uptake may be a health risk because it can cause problems in bone growth [67]. The concentration of Sr in all organs is to some extent comparable and decreased in the order (*μ*g/g): lungs (1.0), kidney (0.6), liver (0.56), muscles (0.42), spleen (0.4), brain (0.34), and heart (0.2) (Table 2).

Exact biological role of Ti is not known [68]. It is believed that Ti is not a poison metal, and the human body can tolerate titanium in large dose. Increased content of titanium was revealed only in lungs of all donors (31 *μ*g/g) and in spleen of two donors (4 and 18 *μ*g/g; Supplementary [Supplementary-material supplementary-material-1]). The detectable amounts of titanium were also found in kidney of these two donors (3.7 and 3.0 *μ*g/g; Supplementary [Supplementary-material supplementary-material-1]).

Thus, for some elements, the biological role and possible reasons for increase in their concentration in some human organs seem to be quite understandable. At the same time, the possible biological role of certain trace elements is not yet completely clear.

In this work, the capability of a high-power two-jet plasma for analysis of twenty elements in seven powdered human organs has been shown. The maximum element concentration were found respectively in the following organs: Al, B, Cr, Ni, Si, Sn, Sr, Ti (lungs), Fe (lungs and spleen), Mn (liver and kidney), Ag, Mo (liver), Ca (lungs and kidney), Cu (brain), Cd (kidney), Pb (brain), and Zn (liver, kidney, and muscle). The minimal content of elements was observed, respectively, in the following organs: Ag (all organs except liver), Ba (spleen, muscles, and brain), Ca and Mg (liver), Si (liver, muscle, and brain), Cd and Sr (heart and brain), Al, Cu, Fe, and Mn (muscle), and Zn (spleen and brain).

## Figures and Tables

**Table 1:** The analysis results of one donor's (number 4) organs (*μ*g/g).

Element	Lung	Spleen	Liver	Kidney	Muscle	Heart	Brain
Ag	0.04^*∗*^	<0.02^*∗∗*^	0.20	0.04	<0.03	<0.02	<0.03
Al	75	2.4	2.1	4.4	1.1	12	2.1
B	1.7	<0.5	0.96	0.96	0.61	0.55	0.59
Ba	4.8	<0.3	0.96	0.40	0.3	0.40	<0.3
Ca	1200	550	310	1330	190	400	430
Cd	7.0	2.2	14	290	0.11	0.24	0.24
Co	0.30	<0.3	<0.2	<0.2	<0.2	<0.2	<0.2
Cr	2.5	<0.5	<0.5	<0.5	<0.5	<0.5	<0.5
Cu	12	9.1	20	19	3.9	15	47
Fe	2900	3930	1200	960	160	420	440
Mg	930	1180	390	1360	890	1100	1260
Mn	1.9	1.2	5.0	10	0.35	0.81	1.8
Mo	0.30	0.87	5.4	3.0	<0.2	<0.2	<0.2
Ni	2.5	<0.5	<0.3	0.83	<0.5	<0.5	<0.5
Pb	<0.3	0.61	<0.5	<0.5	0.64	<0.5	1.9
Si	650	180	93	83	33	84	39
Sn	3.0	<0.5	0.36	<0.5	<0.5	<0.5	<0.3
Sr	0.71	0.28	0.27	0.62	0.22	0.20	0.34
Ti	12	<2	<2	∼2	<2	<2	<2
Zn	180	100	140	540	180	290	120

^*∗*^The relative standard deviation of the results was within 3–12%. ^*∗∗*^The data of two-jet plasma atomic emission spectrometry contained reliable peaks corresponding to some elements, but it was possible to estimate only their approximate concentration.

**Table 2:** The content of different elements in lyophilized preparations of human organs (*μ*g/g).

Element	Average value ± SD or concentration range as well as literature data taken from [46]^*∗*^ are in parentheses						
	Organ						
	Lung^*∗∗*^ (*n* = 6)	Spleen (*n* = 6)	Liver (*n* = 6)	Kidney (*n* = 6)	Muscle (*n* = 5)	Heart (*n* = 6)	Brain (*n* = 1)
Ag	<0.02–0.06	<0.02^*∗∗∗*^	<0.02–0.2	<0.02–0.11	<0.03–0.05	<0.02–0.05	<0.03
Al	263 ± 163 (130 ± 203)^*∗*^	14.5 ± 15.5 (12.5 ± 4.3)	5.9 ± 4.8 (11.3 ± 3.2)	7.4 ± 4.3 (10.4 ± 3.4)	2.2 ± 1.8 (9.4 ± 2.3)	9.2 ± 4.2 (11.4 ± 5.3)	2.1 (13.9 ± 3.2)
B	2.5 ± 1.2	<0.5–1.0	0.8 ± 0.26	0.95 ± 0.50	0.6 ± 0.2	0.57 ± 0.13	0.59
Ba^*β*^	6.9 ± 8.1^ῼ^	<0.3	<0.2–2.8	<0.2–0.40	<0.3–0.3	0.36 ± 0.22	<0.3
Ca	1650 ± 565 (585 ± 205)	511.7 ± 103.0 (335 ± 149)	323 ± 78 (208 ± 121)	1232 ± 410 (529 ± 291)	442 ± 439 (215 ± 90)	413 ± 251 (291 ± 151)	430 (301 ± 187)
Cd	<0.1–7.0 (<4–11.7)	1.3 ± 1.2 (5.4 ± 6.7)	11.5 ± 9.1 (16.7 ± 19.1)	114 ± 122^ῼ^ (237 ± 206)	0.95 ± 1.0^ῼ^ (<0.3–4.8)	<0.1–0.51 (<0.4–7)	0.24 (0.4)
Co	<0.2–0.92 (<0.005–1.4)	<0.3 (<0.001–0.09)	<0.2–1.0 (0.17 ± 0.11)	<0.2–0.38 (0.07 ± 0.1)	<0.2 (<0.001–0.03)	<0.2–0.74 (0.07 ± 0.048)	<0.2 (<0.001–0.07)
Cr	4.0 ± 1.3	<0.5	<0.5	<0.5	<0.5	<0.5	<0.5
Cu	15.2 ± 3.8 (<2.0–38)	10.6 ± 4.8 (<1.5–11)	19.8 ± 5.7 (32 ± 20)	17.5 ± 4.5 (<2.3–45)	4.2 ± 1.4 (<0.8–39)	16.5 ± 6.3 (18.0 ± 5.5)	47 (24 ± 11)
Fe	2900 ± 498 (984 ± 489)	2650 ± 1595 (1400 ± 760)	723 ± 489 (837 ± 522)	825 ± 299 (430 ± 203)	156 ± 18 (136 ± 42)	428 ± 222 (257 ± 80)	440 (225 ± 71)
Mg	1093 ± 161 (<11–1390)	1305 ± 391 (729 ± 216)	603 ± 220 (678 ± 191)	1160 ± 271 (<13–1250)	968 ± 225 (911 ± 153)	1185 ± 517 (1070 ± 210)	1260 (700 ± 244)
Mn	2.7 ± 0.8 (1.17 ± 0.94)	1.3 ± 1.0 (0.82 ± 0.71)	8.5 ± 2.0 (5.58 ± 1.78)	7.9 ± 1.5 (4.92 ± 1.64)	0.47 ± 0.21 (0.74 ± 1.44)	0.86 ± 0.30 (1.63 ± 2.16)	1.8 (1.32 ± 0.49)
Mo	0.33 ± 0.06 (<0.01–2.7)	<0.3–3.7 (<0.37–1.4)	4.9 ± 1.0 (2.1 ± 1.8)	1.9 ± 0.60 (<0.47–2.1)	<0.2 (<0.31–2.2)	<0.2–0.55 (<0.14–3.9)	<0.2 (<0.02–1.65)
Ni	3.1 ± 2.0	<0.5	<0.3	0.82 ± 0.85^ῼ^	<0.5	<0.5	<0.5
Pb	<0.3–0.4	<0.5–0.66	<0.5–0.65	<0.5–1.7	<0.5–0.64	<0.5	1.9
Si	2390 ± 1829	164 ± 107	40.8 ± 29.3	78.2 ± 18.6	31.6 ± 8.3	66.5 ± 40.4	39
Sn	3.2 ± 2.1	<0.5	<0.3–1.1	<0.5	<0.5	<0.5	<0.3
Sr	1.0 ± 0.66	0.40 ± 0.17	0.56 ± 0.52	0.6 ± 0.3	0.42 ± 0.31	0.2 ± 0.09	0.34
Ti	31 ± 17	<2–18	<2	≤2–3.7	<2	<2–2.9	<2
Zn	208 ± 35 (62 ± 19)	156 ± 135 (83 ± 21)	272 ± 153 (228 ± 85)	292 ± 156 (235 ± 70)	290 ± 103 (237 ± 55)	218 ± 92 (126 ± 28)	120 (51 ± 15)

^*∗*^Literature data from [46] obtained by neutron activation analysis are given in parentheses. ^*∗∗*^Element concentrations in 7 different organs were determined by two-jet plasma atomic emission spectrometry; the relative standard deviation of the results from three replicates in the case of each sample analyzed was within 3–12%. The obtained average values for 5-6 samples in the case of seven organs are used to calculate average value ± SD. ^*∗∗∗*^The data of two-jet plasma atomic emission spectrometry contained reliable peaks corresponding to some elements, but it was possible to estimate only their approximate concentration. ^ῼ^In the absence of the Gaussian distribution for several samples, the SD was higher than the average value; in this case, the median (*M*) and interquartile ranges (IQR) were estimated (see Supplementary Tables [Supplementary-material supplementary-material-1]–[Supplementary-material supplementary-material-1]). ^*β*^In one of the donors, the content of Ba in various organs was approximately 3–3000 times higher than in other ones. Therefore, the content of Ba in organs of this donor was not taken into account to calculate the average value and is separately given in Table 3.

**Table 3:** The content of Ba in human organs (*μ*g/g^*∗*^).

Organ	Sample 2	Samples (1, 3–7)	Approximate ratio of values
Lung	5600	1.9–21	267–2950
Liver	130	<0.2–2.8	46->650
Muscle	110	≤0.3^*∗∗*^	≥367
Spleen	39	<0.3	>130
Kidney	3.5	<0.2–0.4	8.8->18
Heart	2.0	0.16–0.7	2.9–13

^*∗*^Barium concentration in 1–7 samples was determined by two-jet plasma atomic emission spectrometry; the relative standard deviation of the results from three replicates was within 3–8%. ^*∗∗*^The data of two-jet plasma atomic emission spectrometry contained reliable peaks corresponding to some elements, but it was possible to estimate only their approximate concentration.

## Data Availability

All data used to support the findings of this study are included within the article.

## Abbreviations

AAS: Atomic absorption spectrometry
CRMs: Certified reference materials
ICP-MS: Inductively coupled plasma mass spectrometry
ICP-AES: Coupled inductively plasma atomic emission spectrometry
NAA: Neutron activation analysis
TJP-AES: Two-jet plasma atomic emission spectrometry.

## Supplementary Materials

Supplementary Materials Supplementary Tables 1–5: information concerning the content of different elements in different organs.
