# Supplementary material for: Twenty Element Concentrations in Human Organs Determined by Two-Jet Plasma Atomic Emission Spectrometry
Source: ScientificWorldJournal. 2019 Oct 24;2019:9782635. doi: 10.1155/2019/9782635 (PMC6854933; doi:10.1155/2019/9782635)
Supplement: Supplementary Materials — Supplementary Tables 1–5: information concerning the content of different elements in different organs. [file 9782635.f1.pdf]

Supplementary Table 1. The content of different elements in lyophilized human spleen preparations (μg/g)*.

Element	Number of spleen preparation						Average value ± S.D. (or range)	Median and in parenthesis interquartile range
	1	2	3	4	5	6		
Ag	<0.02[Ω]	<0.02	<0.02	<0.02	<0.02	<0.02	<0.02	NP[μ]
Al	34	35	4.9	2.4	5.9	4.9	14.5±15.5**	5.4 (29.1)
B	1.0	0.79	1.0	<0.6	<0.6	<0.6	(<0.6–1.0)	NP
**B**a	<0.3	39***	<0.3	<0.3	<0.3	<0.3	<0.3***	NP
Ca	560	390	480	550	420	670	511.7±103.0	515 (140)
Cd	0.98	3.0	0.11	2.2	0.1	1.5	1.3±1.2	1.24 (2.09)
Co	<0.3	<0.3	<0.3	<0.3	<0.3	<0.3	<0.3	NP
Cr	<0.5	<0.5	<0.5	<0.5	<0.5	<0.5	<0.5	NP
Cu	11	8.3	7.7	9.1	7.6	20	10.6±4.8	8.7 (3.3)
Fe	710	880	2250	3930	4150	3980	2650±1595	3090 (3100)
Mg	1150	1400	810	1180	1300	1990	1305±391	1240 (250)
Mn	0.83	0.75	0.77	1.2	1.0	3.3	1.3±1.0	0.915 (0.43)
Mo	<0.3	<0.3	0.3	0.87	0.63	3.7	(<0.3–3.7)	NP
Ni	<0.5	<0.5	<0.5	<0.5	<0.5	<0.5	<0.5	NP
Pb	<0.5	<0.5	<0.5	0.61	<0.5	0.66	(<0.5–0.66)	NP
Si	360	180	77	180	76	110	164±107	145 (103)
Sn	<0.5	<0.5	<0.5	<0.5	<0.5	<0.5	<0.5	NP
Sr	0.43	0.72	0.38	0.28	0.29	0.28	0.40±0.17	0.335 (0.15)
Ti	4.0	18	<2	<2	<2	<2	(<2–18)	NP
Zn	100	130	84	100	89	430	156±135	100 (41)

*Element concentrations were determined by two-jet plasma atomic emission spectrometry; the relative standard deviation of the results from three replicates was within 3-12 %.
**In the absence of the Gaussian distribution, in some cases the S.D. values exceeded the average values. Taking this into account for values of Al, median (M) and interquartile ranges (IQR) were also estimated: M = 5.4 , IQR = 29.1
***Several sample sets did not fit the Gaussian distribution. Therefore, the analysis results of the sample 2 were not taken into account. For this donor, the barium content in different organs was given separately (Table 2).
[Ω]The data of two-jet plasma atomic emission spectrometry contained reliable peaks corresponding to some elements, but it was possible to estimate only their approximate concentration.
[μ]NP – no possible: it was no possible to calculate Median and interquartile range since of the availability of only approximate data.

Supplementary Table 2. The content of different elements in lyophilized human liver preparations (µg/g)*.

Element	Number of liver preparation						Average value ± S.D. (or range)	Median and in parenthesis interquartile range
	1	2	3	4	5	6		
Ag	0.20	0.05	<0.02[Ω]	0.20	0.20	<0.02	(<0.02–0.2)	NP[µ]
Al	12	12	3.9	2.1	2.1	3.3	5.9±4.8	3.6 (9.9)
B	0.40	0.61	1.1	0.96	0.87	0.91	0.8±0.26	0.89 (0.35)
Ba	<0.2	130**	2.8	0.96	0.20	<0.2	(<0.2–2.8)**	NP
Ca	430	240	410	310	270	280	323±78	295 (140)
Cd	18	25	6.3	14	1.1	4.8	11.5±9.1	10.15 (13.2)
Co	1.0	0.61	<0.2	<0.2	<0.2	0.4	(<0.2–1.0)	0.61 (0.6)
Cr	<0.5	<0.5	<0.5	<0.5	<0.5	<0.5	<0.5	NP
Cu	12	16	19	20	28	24	19.8±5.7	19.5 (8)
Fe	280	670	310	1200	1200	1400	723±489	935 (890)
Mg	320	640	590	390	870	810	603±220	615 (420)
Mn	9.1	9.6	7.5	5.0	10	10	8.5±2.0	9.35 (2.5)
Mo	6.6	3.7	4.7	5.4	4.7	4.5	4.9±1.0	4.7 (0.9)
Ni	<0.3	<0.3	<0.3	<0.3	<0.3	<0.3	<0.3	NP
Pb	<0.5	<0.5	<0.5	<0.5	<0.5	0.65	(<0.5–0.65)	NP
Si	58	22	26	93	17	29	40.8±29.3	27.5 (36)
Sn	0.78	<0.3	0.41	0.36	0.3	1.1	<0.3–1.1)	0.41 (0.42)
Sr	0.47	1.6	0.41	0.27	0.27	0.33	0.56±0.52	0.37 (0.2)
Ti	<2	<2	<2	<2	<2	<2	<2	NP
Zn	140	290	170	140	370	520	272±153	230 (230)

*Element concentrations were determined by two-jet plasma atomic emission spectrometry; the relative standard deviation of the results from three replicates was within 5-12 %.
**Several sample sets did not fit the Gaussian distribution. Therefore, the analysis results of the sample 2 (Ba) were not taken into account to calculate the average value. For one of the donors (number 2), the barium content in various organs was given separately (Table 2).
[Ω]The data of two-jet plasma atomic emission spectrometry contained reliable peaks corresponding to some elements, but it was possible to estimate only their approximate concentration.
[µ]NP –no possible: it was no possible to calculate Median and interquartile range since of the availability of only approximate data.

Supplementary Table 3. The content of different elements in lyophilized human kidney preparations (μg/g)*.

Element	Number of kidney preparation						Average value ± S.D. (or range)	Median and in parenthesis interquartile range
	1	2	3	4	5	6		
Ag	0.11	<0.02[Ω]	<0.02	0.04	<0.02	<0.02	<0.02–0.11)	NP[μ]
Al	7.2	16	5.3	4.4	5.9	5.7	7.4±4.3	5.8 (1.9)
B	0.80	0.71	1.9	0.96	0.84	0.46	0.95±0.50	0.82 (0.25)
Ba	0.40	3.5**	0.35	0.40	0.2	0.40	(0.2–0.40)**	0.4 (0.05)
Ca	1950	1310	960	1330	1060	780	1232±410	1185 (370)
Cd	59	250	41	290	9.3	37	114±122***	50 (213)
Co	0.38	<0.2	<0.2	<0.2	<0.2	<0.2	<0.2–0.38	NP
Cr	<0.5	<0.5	<0.5	<0.5	<0.5	<0.5	<0.5	NP
Cu	21	15	13	19	13	24	17.5±4.5	17 (8)
Fe	460	490	1180	960	800	1060	825±299	880 (570)
Mg	1300	1260	750	1360	890	1400	1160±271	1280 (470)
Mn	8.5	7.9	7.4	10	5.5	8.1	7.9±1.5	8 (1.1)
Mo	2.1	1.5	1.7	3.0	1.1	1.9	1.9±0.60	1.8 (0.6)
Ni	2.5	0.34	0.33	0.83	0.36	0.53	0.82±0.85***	0.445 (0.49)
Pb	1.7	<0.5	<0.5	<0.5	<0.5	<0.5	(<0.5-1.7)	NP
Si	80	110	66	83	55	75	78.2±18.6	77.5 (17)
Sn	<0.5	<0.5	<0.5	<0.5	<0.5	<0.5	<0.5	NP
Sr	1.2	0.48	0.42	0.62	0.45	0.5	0.6±0.3	0.49 (0.17)
Ti	3.7	3.0	~2	~2	~2	~2	(~2 – 3.7)	NP
Zn	230	420	140	540	170	250	292±156	240 (250)

*Element concentrations were determined by two-jet plasma atomic emission spectrometry; the relative standard deviation of the results from three replicates was within 3-12 %.
**Several sample sets did not fit the Gaussian distribution. Therefore, the analysis results of the sample 2 (Ba) were not taken into account to calculate the average value. The barium content in various organs of the second donor was given separately (Table 2).
***In the absence of the Gaussian distribution, in some cases when the S.D. values exceeded the average ones, the median (M) and interquartile ranges (IQR) were also estimated: Cd: M = 50, IQR = 213 ; Ni: M = 0.44, IQR = 0.49.
[Ω]The data of two-jet plasma atomic emission spectrometry contained reliable peaks corresponding to some elements, but it was possible to estimate only their approximate concentration.
[μ]NP – no possible: it was no possible to calculate Median and interquartile range since of the availability of only approximate data.

Supplementary Table 4. The content of different elements in five lyophilized preparations of human muscles (μg/g)*.

Element	Number of muscle preparation					Average value ± S.D. (or range)	Median and in parenthesis interquartile range
	1	2	3	4	5		
Ag	<0.03[Ω]	0.05	<0.03	<0.03	<0.03	(<0.03–0.05)	NP[μ]
Al	2.5	5.2	0.81	1.1	1.5	2.2±1.8	1.5 (1.4)
B	0.84	0.50	0.50	0.61	0.30	0.6±0.2	0.5 (0.11)
Ba	<0.3	110**	0.3	0.3	<0.3	(< 0.3–0.3)**	NP
Ca	1220	330	200	190	270	442±439***	270 (130)
Cd	1.7	2.4	0.25	0.11	0.27	0.95±1.0***	0.27 (1.45)
Co	<0.2	<0.2	<0.2	<0.2	<0.2	<0.2	NP
Cr	<0.5	<0.5	<0.5	<0.5	<0.5	<0.5	NP
Cu	6.1	3.7	2.5	3.9	4.9	4.2±1.4	3.9 (1.2)
Fe	150	130	160	160	180	156±18	160 (10)
Mg	1100	1030	620	890	1200	968±225	1030 (210)
Mn	0.75	0.60	0.21	0.35	0.44	0.47±0.21	0.44 (0.25)
Mo	<0.2	<0.2	<0.2	<0.2	<0.2	<0.2	NP
Ni	<0.5	<0.5	<0.5	<0.5	<0.5	<0.5	NP
Pb	<0.3	<0.3	<0.3	0.64	<0.5	(<0.5–0.64)	NP
Si	45	28	23	33	29	31.6±8.3	29 (5)
Sn	<0.5	<0.5	<0.5	<0.5	<0.5	<0.5	NP
Sr	0.65	0.85	0.15	0.22	0.21	0.42±0.31	0.22 (0.44)
Ti	<2	<2	<2	<2	<2	<2	NP
Zn	420	340	190	180	320	290±103	320 (150)

*Element concentrations were determined by two-jet plasma atomic emission spectrometry; the relative standard deviation of the results from three replicates was within 3-12 %.
**Several sample sets did not fit the Gaussian distribution. Therefore, the analysis results of the sample 2 were not taken into account to calculate the average value. The barium content in various organs of the second donor was given separately (Table 2).
***In the absence of the Gaussian distribution, in some cases when the S.D. values exceeded or comparable with the average ones, the median (M) and interquartile ranges (IQR) were also estimated: Ca: M = 270, IQR = 130 ; Cd: M = 0.3 , IQR = 1.4
[Ω]The data of two-jet plasma atomic emission spectrometry contained reliable peaks corresponding to some elements, but it was possible to estimate only their approximate concentration.
[μ]NP –no possible: it was no possible to calculate Median and interquartile range since of the availability of only approximate data.

Supplementary Table 5. The content of different elements in lyophilized human heart preparations (µg/g)*.

Element	Number of heart preparation						Average value ± S.D. (or range)	Median and in parenthesis interquartile range
	1	2	3	4	5	6		
Ag	0.05	<0.02[Ω]	<0.02	<0.02	<0.02	<0.02	(<0.02–0.05)	NP[µ]
Al	16	8.0	4.2	12	6.3	8.4	9.2 ±4.2	8.2 (5.7)
B	0.78	0.40	0.65	0.55	0.57	0.48	0.57 ±0.13	0.56 (0.17)
Ba	0.70	2.0**	0.17	0.40	0.35	0.16	0.36±0.22**	0.375 (0.53)
Ca	780	220	130	400	310	640	413 ±251	355 (420)
Cd	0.51	0.18	0.16	0.24	0.1	0.1	(0.1–0.51)	0.17 (0.14)
Co	0.74	0.48	<0.2	<0.2	<0.2	<0.2	(<0.2–0.74)	NP
Cr	<0.5	<0.5	<0.5	<0.5	<0.5	<0.5	<0.5	NP
Cu	24	18	5.7	15	15	21	16.5 ±6.3	16.5 (6)
Fe	420	260	210	420	420	840	428±222	420 (160)
Mg	1900	1100	440	1100	940	1630	1185±517	1100 (690)
Mn	1.2	0.63	0.42	0.81	1.1	1.0	0.86±0.30	0.905 (0.47)
Mo	0.55	0.25	<0.2	<0.2	0.25	0.32	(<0.2–0.55)	0.285 (0.185)
Ni	<0.5	<0.5	<0.5	<0.5	<0.5	<0.5	<0.5	NP
Pb	<0.5	<0.5	<0.5	<0.5	<0.5	<0.5	<0.5	NP
Si	140	28	55	84	44	48	66.5±40.4	51.5 (40)
Sn	<0.5	<0.5	<0.5	<0.5	<0.5	<0.5	<0.5	NP
Sr	0.37	0.13	0.1	0.20	0.19	0.20	0.2±0.09	0.195 (0.07)
Ti	<2	<2	2.9	<2	<2	<2	(<2–2.9)	NP
Zn	330	210	75	290	160	240	218±92	225 (130)

*Element concentrations were determined by two-jet plasma atomic emission spectrometry; the relative standard deviation of the results from three replicates was within 3-12 %.
**Several sample sets did not fit the Gaussian distribution. Therefore, the analysis results of the sample 2 were not taken into account to calculate the average value. The barium content in various organs of the second donor was given separately (Table 2).
[Ω]The data of two-jet plasma atomic emission spectrometry contained reliable peaks corresponding to some elements, but it was possible to estimate only their approximate concentration.
[µ]NP –no possible: it was no possible to calculate Median and interquartile range since of the availability of only approximate data.

Supplementary Table 6. The content of different elements in lyophilized preparation of human lungs (μg/g)*.

Element	Number of lung preparation						Average value ± S.D. (or range)	Median and in parenthesis interquartile range
	1	2	3	4	5	6		
Ag	0.06	<0.02[Ω]	<0.02	0.04	<0.02	<0.02	(<0.02–0.06)	NP[μ]
Al	340	230	530	75	290	110	263±163	260 (230)
B	3.0	1.8	4.6	1.7	2.0	1.7	2.5±1.2	1.9 (1.3)
Ba	5.4	5600**	21	4.8	1.5	1.9	6.9±8.1***	5.1 (19.1)
Ca	2700	1800	1200	1200	1600	1400	1650±565	1500 (600)
Cd	1.0	4.3	1.9	7.0	0.11	0.1	(0.1–7.0)	1.45 (4.19)
Co	0.92	0.81	0.74	0.30	0.2	0.2	(0.2–0.92)	0.52 (0.61)
Cr	5.0	5.5	4.7	2.5	3.5	2.7	4.0±1.3	4.1 (2.3)
Cu	19	13	13	12	13	21	15.2±3.8	13 (6)
Fe	3400	2400	2600	2900	2500	3600	2900±498	2750 (900)
Mg	1150	1300	1100	930	880	1200	1093±161	1125 (270)
Mn	3.5	3.5	3.4	1.9	2.3	1.8	2.7±0.82	2.85 (1.6)
Mo	0.41	0.24	0.31	0.30	0.39	0.32	0.33±0.06	0.315 (0.090)
Ni	6.7	3.1	3.5	2.5	0.85	2.0	3.1±2.0	2.8 (1.5)
Pb	0.41	<0.3	<0.3	<0.3	<0.3	<0.3	(<0.3–0.41)	NP
Si	3100	1600	5600	650	2500	890	2390±1829	2050 (2210)
Sn	2.0	2.2	6.1	3.0	5.2	0.5	3.2±2.1	2.6 (3.2)
Sr	2.1	31**	1.2	0.71	0.47	0.66	1.0±0.66	0.955 (1.44)
Ti	42	30	54	12	37	12	31±17	33.5 (30)
Zn	230	260	170	180	185	220	208±35	202.5 (50)

*Element concentrations were determined by two-jet plasma atomic emission spectrometry; the relative standard deviation of the results from three replicates was within 3-12 %.
**Several sample sets did not fit the Gaussian distribution. Therefore, the analysis results of the sample 2 (Ba and Sr) were not taken into account to calculate the average value. The barium content in various organs of the second donor was given separately (Table 2).
***Even without taking into account the barium content of the second donor, S.D. was larger than the average value; the median (M) and interquartile ranges (IQR) were also estimated: Ba: M = 4.8, IQR = 3.5.
[Ω]The data of two-jet plasma atomic emission spectrometry contained reliable peaks corresponding to some elements, but it was possible to estimate only their approximate concentration.
